# Preoperative systemic immune‐inflammation index as a significant prognostic factor after TURBT in patients with non‐muscle‐invasive bladder cancer: A retrospective study based on propensity score matching analysis

**DOI:** 10.1002/cam4.5501

**Published:** 2022-12-07

**Authors:** Li Ding, Xiangbu Wang, Xiaobin Deng, Wentao Xia, Kun Wang, Xianlin Yu, Yaotian Huang, Junqi Wang

**Affiliations:** ^1^ Department of Urology The Affiliated Hospital of Xuzhou Medical University Xuzhou PR China; ^2^ Department of Pathology The Affiliated Hospital of Xuzhou Medical University Xuzhou PR China; ^3^ Department of Urology The First Affiliated Hospital of Guangxi Medical University Nanning PR China

**Keywords:** bladder cancer, NMIBC, prognosis, propensity score matching, systemic immune‐inflammation index, tumor recurrence

## Abstract

**Objective:**

To investigate the association of the preoperative systemic immune‐inflammation index (SII) with recurrence‐free survival (RFS) after transurethral resection of the bladder tumor (TURBT) of non‐muscle‐invasive bladder cancer (NMIBC) using propensity score matching (PSM) analysis.

**Methods:**

The clinicopathological characteristics and follow‐up data of NMIBC patients were collected retrospectively from two tertiary medical centers. A 1:1 PSM analysis was carried out using the nearest‐neighbor method (caliper size: 0.02). Cox regression analysis was used to identify the risk factors associated with RFS.

**Results:**

A total of 416 NMIBC patients were included in this study. Before and after matching, patients with increased SII had worse RFS (*p* < 0.0001 and *p* = 0.027, respectively). Multivariate Cox analysis identified SII as an independent predictor of RFS before (HR [95% CI]: 1.789 [1.232, 2.599], *p* = 0.002) and after matching (HR [95% CI]: 1.646 [1.077, 2.515], *p* = 0.021). In the matched subgroup analysis, an elevated SII had a significant association with postoperative worse RFS in the T1 stage (*p* = 0.025), primary status (*p* = 0.049), high‐grade (*p* = 0.0015), and multiple lesions (*p* = 0.043) subgroups.

**Conclusion:**

SII could accurately stratify the prognosis of NMIBC patients before and after PSM analysis. An elevated SII was significantly associated with worse RFS in NMIBC patients.

## Abstract 2

Kaplan–Meier analysis for recurrence‐free survival in subgroups based on the systemic immune‐inflammation index (SII) after propensity score matching. SII accurately stratified between high and low risk groups of poor RFS in the T1 stage (*p* = 0.025), primary status (*p* = 0.049), high grade (*p* = 0.0015), and multiple lesion (*p* = 0.043) subgroups.

## 1 INTRODUCTION

Bladder cancer (BC) is one type of prevalent urological malignancy with a high morbidity, with nearly 573,278 new cases and 212,536 associated deaths reported worldwide.^1^, ^2^ The most common pathological type of BC is urothelial carcinoma. Once BC progresses to muscle‐invasive bladder cancer (MIBC), most patients will undergo radical cystectomy, resulting in a poor quality of life.^3^, ^4^ However, even after receiving the standard treatment regimen, most MIBC patients still have a poor prognosis. Meanwhile, approximately 75% of BC cases are non‐muscle invasive bladder cancer (NMIBC) when initially diagnosed, and even after receiving transurethral resection of bladder tumor(TURBT), there is a high chance of BC tumor recurrence and progression to MIBC.^5^, ^6^, ^7^ The models for the evaluation of patients with NMIBC that are now widely accepted clinically are mainly from the European Organisation for Research and Treatment of Cancer (EORTC)^3^, ^4^ and the Spanish Urological Organization (Club Urologico Español de Tratamiento Oncologico, CUETO),^5^ and numerous real‐world validation studies have demonstrated the feasibility of these models.^6^, ^7^ Whereas, optimization of the existing models is needed, and mining novel valuable variables can further improve the performance of the models.

Hematological indicators can be used to assess the hypothesis that inflammation and nutritional status are important contributors in tumor development and progression in several investigations.^8^, ^9^, ^10^, ^11^, ^12^, ^13^ Previous studies have proposed several novel indicators based on inflammatory and nutrition‐related hematological parameters, often referred to as systemic inflammatory response (SIR) indicators. These markers offer a lot of potential for clinical use as they are rapid, precise, practical, and affordable. The systemic immune‐inflammation index (SII) has been proposed as a potential SIR indicator that may be helpful for prognostic prediction in patients with various malignancies.^14^, ^15^, ^16^ However, studies examining its value in NMIBC are still limited.

As a model visualization method, nomogram has been widely used as prognostic models for various diseases.^17^, ^18^, ^19^, ^20^ This study aimed to evaluate the real‐world value of SII for predicting RFS after TURBT in NMIBC patients using propensity score matching (PSM) analysis and eventually construct prediction models based on statistically significant variables.

## 2 MATERIALS AND METHODS

### 2.1 Study population

This study was approved by the Ethics Committee of the Affiliated Hospital of Xuzhou Medical University and the Ethics Committee of the First Affiliated Hospital of Guangxi Medical University. Retrospective data collection on patients having a pathological diagnosis of urothelial carcinoma of the bladder between October 2018 and June 2021 was performed using the medical record databases of two tertiary medical hospitals in China. Inclusion criteria were: (1) primary lesion in the bladder; (2) complete surgical records and surgical approach; (3) complete postoperative pathology report; (4) clear imaging without regional lymph nodes or distant metastases. Exclusion criteria were: (1) having multiple cancers (2) tumor recurrence, progression or death occurred within 1 month; (3) clinical, laboratory and follow‐up data incomplete. The pathological information, including the T category and pathology grade, was re‐evaluated by one experienced pathologist. SII was defined as platelet count x neutrophil count/lymphocyte count (109/L). The time from TURBT to the first evidence of either recurrent (or progression), cancer‐related death, or last follow‐up was referred to as recurrence‐free survival (RFS).

### 2.2 Statistical analysis

SII was transformed from continuous to categorical variables (cut‐off value = 505) using the X‐tile program.^22^ Continuous data were presented as mean with standard deviation (SD), median with interquartile range (IQR), and categorical data as numbers (percentages). The Chi‐square test was used for categorical variables, and the Mann–Whitney U test was used for non‐normally distributed continuous variables. A 1:1 PSM analysis was carried out to balance the baseline characteristics using the nearest‐neighbor method with a caliper size of 0.02. The clinical endpoints of patients were determined using the Kaplan–Meier method and analyzed using the log‐rank test. The univariate and multivariate Cox regression analyses were used to calculate the hazard ratio (HR) with a 95% confidence interval (CI) to identify risk factors. The nomogram model validation was performed using the area under the receiver operating characteristics curve (AUC) for discrimination ability and calibration curves for calibration ability. X‐tile 3.6.1 (http://tissuearray.org/), SPSS 26.0 (IBM Corp.), and R 4.1.2 (http://www.R‐project.org/) were used to analyze the database statistically. A two‐sided value of *p* < 0.05 was considered statistically significant.

## 3 RESULTS

### 3.1 Patient characteristics

A total of 416 patients from two tertiary medical centers were included in this study, with 299 patients from the Affiliated Hospital of Xuzhou Medical University and 117 from the First Affiliated Hospital of Guangxi Medical University. The characteristics of patients before and after matching are detailed in Table 1. The median (IQR) follow‐up time was 21 (14.75–32) months, and the median (IQR) age at diagnosis was 67 (58–75) years, with 354 (85.096%) males. Moreover, 169 (40.625%) patients were overweight. Most of the patients (79.808%) were presented with painless gross hematuria. The median (IQR) maximum tumor diameter was 20 (14–30) mm. Most tumors were in the Ta stage (59.615%), with primary status (86.298%), high pathology grade (58.413%), and multiple lesions (55.288%). A more balanced cohort was constructed with 1:1 matching of PSM according to SII, with 141 subjects in each of the two groups.

**TABLE 1:** Baseline characteristics of study population before and after propensity score matching

Variables	Level	Before propensity matching				After propensity matching			
		Total	SII < 505	SII ≥505	*p*	Total	SII < 505	SII ≥505	*p*
		*N* = 416	*N* = 264	*N* = 152		*N* = 282	*N* = 141	*N* = 141	
Age (years), median [IQR]		67 [58, 75]	66 [58, 74]	67 [59, 75]	0.374	66 [58, 75]	65 [58, 73]	67 [59, 75]	0.405
Maximum tumor diameter (mm), median [IQR]		20 [14, 30]	20 [14, 30]	20 [15, 30]	0.399	20 [15, 30]	20 [14, 30]	20 [15, 30]	0.861
Gender, *n* (%)	Female	62 (14.904)	41 (15.530)	21 (13.816)	0.636	39 (13.830)	19 (13.475)	20 (14.184)	0.863
	Male	354 (85.096)	223 (84.470)	131 (86.184)		243 (86.170)	122 (86.525)	121 (85.816)	
BMI, *n* (%)	<25	247 (59.375)	151 (57.197)	96 (63.158)	0.233	175 (62.057)	89 (63.121)	86 (60.993)	0.713
	≥25	169 (40.625)	113 (42.803)	56 (36.842)		107 (37.943)	52 (36.879)	55 (39.007)	
Gross hematuria, *n* (%)	No	84 (20.192)	51 (19.318)	33 (21.711)	0.558	56 (19.858)	28 (19.858)	28 (19.858)	1
	Yes	332 (79.808)	213 (80.682)	119 (78.289)		226 (80.142)	113 (80.142)	113 (80.142)	
T category, *n* (%)	Ta	248 (59.615)	166 (62.879)	82 (53.947)	0.074	156 (55.319)	78 (55.319)	78 (55.319)	1
	T1	168 (40.385)	98 (37.121)	70 (46.053)		126 (44.681)	63 (44.681)	63 (44.681)	
Prior recurrence status, *n* (%)	Primary	359 (86.298)	234 (88.636)	125 (82.237)	0.068	237 (84.043)	118 (83.688)	119 (84.397)	0.871
	Recurrent	57 (13.702)	30 (11.364)	27 (17.763)		45 (15.957)	23 (16.312)	22 (15.603)	
Pathology grade, *n* (%)	Low‐grade	173 (41.587)	108 (40.909)	65 (42.763)	0.712	124 (43.972)	64 (45.390)	60 (42.553)	0.631
	High‐grade	243 (58.413)	156 (59.091)	87 (57.237)		158 (56.028)	77 (54.610)	81 (57.447)	
Tumor number, *n* (%)	Single	186 (44.712)	124 (46.970)	62 (40.789)	0.222	128 (45.390)	69 (48.936)	59 (41.844)	0.232
	Multiple	230 (55.288)	140 (53.030)	90 (59.211)		154 (54.610)	72 (51.064)	82 (58.156)	

Abbreviations: BMI, body mass index = weight/height^2^; IQR, inter‐quartile range; *N*, number of patients; SII, systemic immune‐inflammation index.

Bolded *p*‐values indicate statistically significant correlations (*p* < 0.05).

### 3.2 Prognostic values of the systemic immune‐inflammation index

**FIGURE 1:**
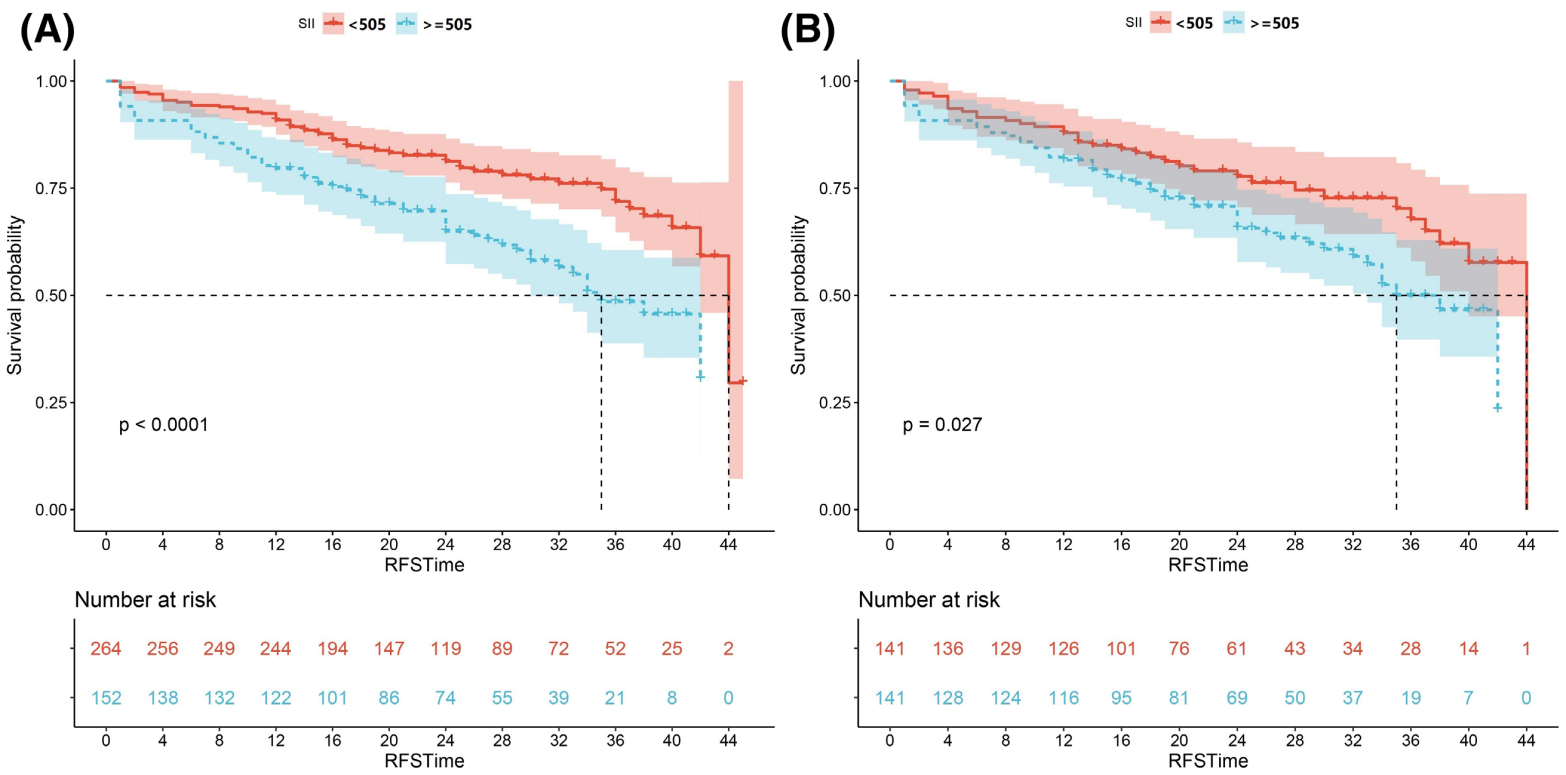
Kaplan–Meier analysis for recurrence‐free survival based on the systemic immune‐inflammation index (SII) in (A) all patients and (B) patients after propensity score matching.

The Kaplan–Meier survival analysis (Figure 1) showed that the patients in the high SII group had significantly worse RFS than the patients in the low SII group (*p* < 0.0001 before PSM and *p* = 0.027 after PSM).

### 3.3 Subgroup analysis before propensity score matching

**FIGURE 2:**
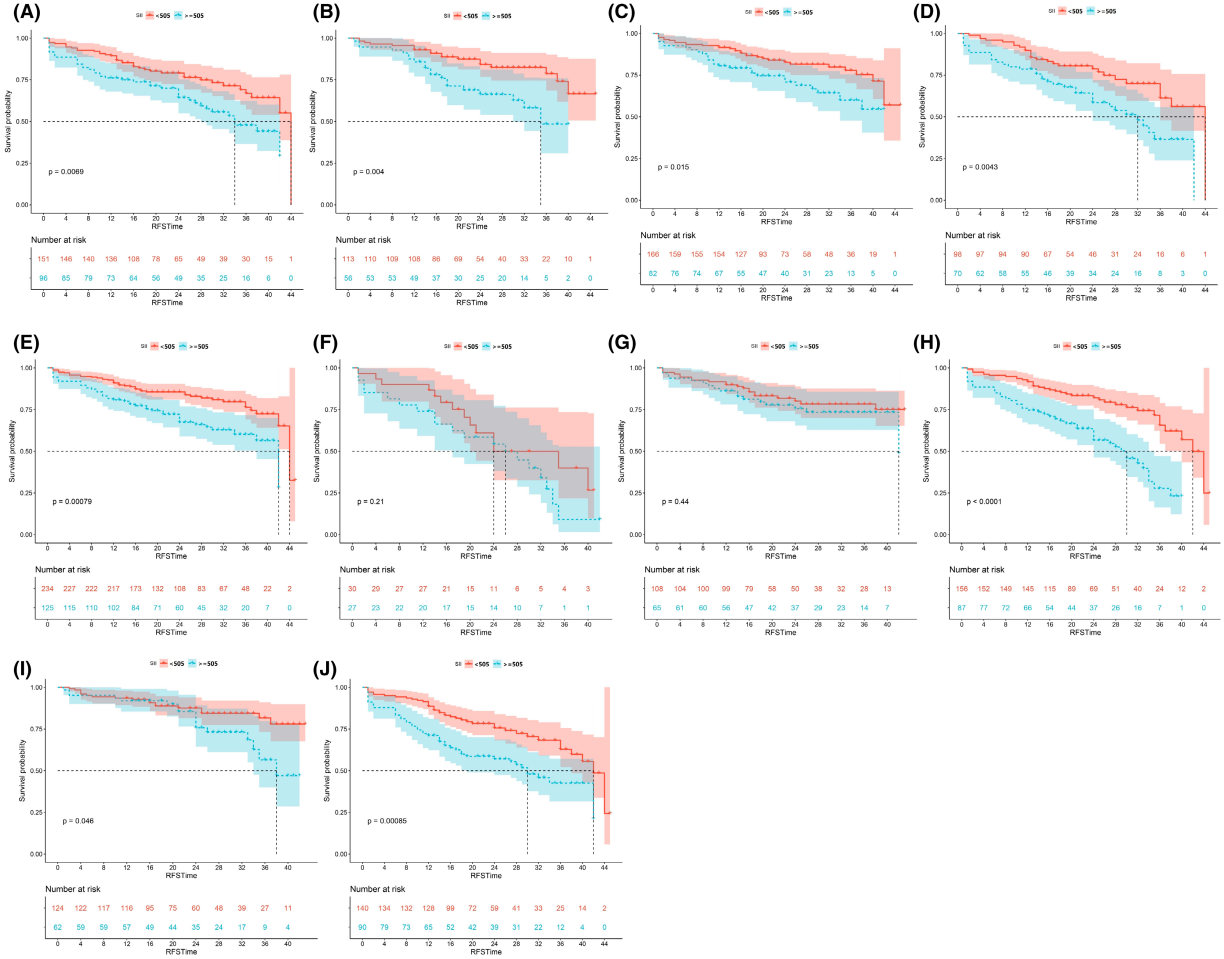
Kaplan–Meier analysis for recurrence‐free survival in subgroups based on the systemic immune‐inflammation index (SII) before propensity score matching. (A) RFS in the non‐overweight subgroup; (B) RFS in the overweight subgroup; (C) RFS in the Ta stage subgroup; (D) RFS in the T1 stage subgroup; (E) RFS in the primary status subgroup; (F) RFS in the recurrent status subgroup; (G) RFS in the low‐grade subgroup; (H) RFS in the high‐grade subgroup; (I) RFS in the single lesion subgroup; (J) RFS in the multiple lesions subgroup. RFS, recurrence‐free survival.

Before PSM, SII accurately stratified between high and low risk groups of poor RFS in non‐overweight (*p* = 0.0069), overweight (*p* = 0.0004), Ta stage (*p* = 0.015), T1 stage (*p* = 0.0043), primary status (*p* = 0.00079), high grade (*p* < 0.0001), single lesion (*p* = 0.046), and multiple lesion (*p* = 0.00085) subgroups (Figure 2).

### 3.4 Subgroup analysis after propensity score matching

**FIGURE 3:**
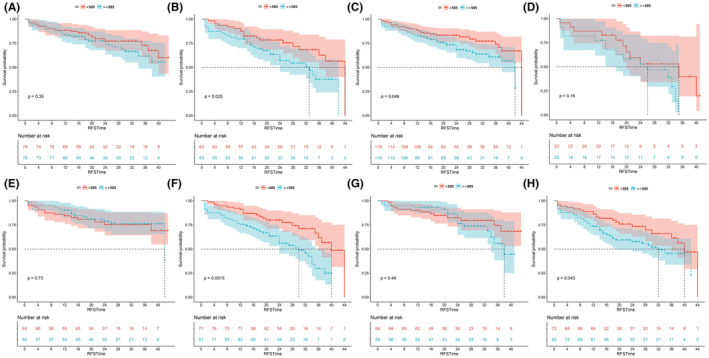
Kaplan–Meier analysis for recurrence‐free survival in subgroups based on the systemic immune‐inflammation index (SII) after propensity score matching. (A) RFS in the Ta stage subgroup; (B) RFS in the T1 stage subgroup; (C) RFS in the primary status subgroup; (D) RFS in the recurrent status subgroup; (E) RFS in the low‐grade subgroup; (F) RFS in the high‐grade subgroup; (G) RFS in the single lesion subgroup; (H) RFS in the multiple lesions subgroup. RFS, recurrence‐free survival.

After PSM, SII accurately stratified between high and low risk groups of poor RFS in the T1 stage (*p* = 0.025), primary status (*p* = 0.049), high grade (*p* = 0.0015), and multiple lesion (*p* = 0.043) subgroups (Figure 3).

### 3.5 Recurrence‐free survival prediction models for NMIBC patients

Before PSM, the univariable analysis showed that elevated SII (HR [95% CI]: 2.039 [1.422, 2.924], *p* < 0.001) was a risk factor for the RFS of NMIBC patients (Figure 2). Furthermore, the T category, prior recurrence status, pathology grade, and tumor number were statistically significant predictors for RFS (*p* < 0.05). After performing PSM adjustment (Table 3), SII, prior recurrence status, pathology grade, and tumor number were suggested to be lined to RFS (all *p* < 0.05). Notably, BMI before PSM (HR [95% CI]: 0.684 [0.467, 1.003], *p* = 0.052) and the T category after PSM (HR [95% CI]: 1.478 [0.978, 2.234], *p* = 0.063) were found to be potential factors for patients' RFS. They were included in the multivariable analysis to investigate their predictive values further.

**FIGURE 4:**
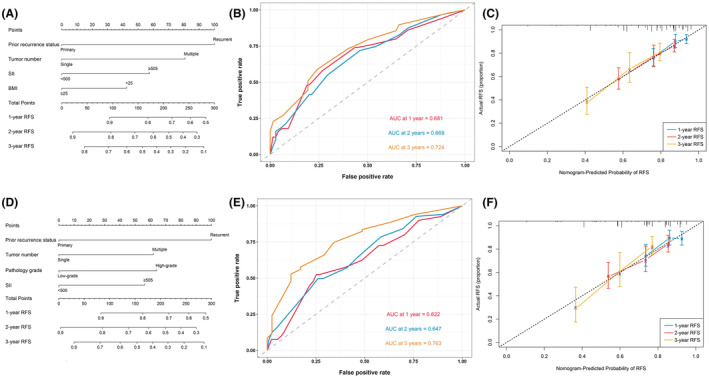
(A) The nomogram for predicting RFS in NMIBC patients before PSM. (B) Time‐dependent ROC curves of the nomogram before PSM. (C) Calibration plot of the nomogram by bootstrapping with 1000 resamples predicting RFS before PSM. (D) The nomogram for predicting RFS in NMIBC patients after PSM. (E) Time‐dependent ROC curves of the nomogram after PSM. (F) Calibration plot of the nomogram by bootstrapping with 1000 resamples predicting RFS after PSM. RFS, recurrence‐free survival; NMIBC, non‐muscle‐invasive bladder cancer; PSM, propensity score matching; ROC, receiver operating characteristic; AUC, area under curve; SII, systemic immune‐inflammation index.

Multivariable analysis results revealed that before PSM (Table 2), SII (HR [95% CI]: 1.789 [1.232, 2.599], *p* = 0.002), BMI (HR [95% CI]: 0.649 [0.441, 0.957], *p* = 0.029), prior recurrence status (HR [95% CI]: 2.641 [1.753, 3.979], *p* < 0.001), and tumor number (HR [95% CI]: 2.006 [1.340, 3.003], *p* < 0.001) remained as independent prognostic factors. After PSM (Table 3), SII (HR [95% CI]: 1.646 [1.077, 2.515], *p* = 0.021), prior recurrence status (HR [95% CI]: 2.475 [1.563, 3.918], *p* < 0.001), pathology grade (HR [95% CI]: 1.703 [1.038, 2.795], *p* = 0.035), and tumor number (HR [95% CI]: 1.738 [1.116, 2.705], *p* = 0.014) were considered as independent prognostic factors. The nomogram models were constructed based on the significant relative variables above (Figure 4A,D). The ROC curves showed the nomogram models' performances regarding 1‐, 2‐, and 3‐year RFS rates (Figure 4B,E). The calibration plots validated by 1000 bootstrap resampling proved the appreciable reliability of the nomogram models (Figure 4C,F).

**TABLE 2:** Univariable and multivariable Cox regression analysis before propensity score matching

Variables	Univariable Cox analysis			Multivariable Cox analysis		
	HR	95% CI	*p*	HR	95% CI	*p*
Age (years)	0.997	[0.984, 1.011]	0.715			
Maximum tumor diameter (mm)	1.007	[0.993, 1.022]	0.314			
SII						
<505	1 (reference)			1 (reference)		
≥505	2.039	[1.422, 2.924]	**<0.001**	1.789	[1.232, 2.599]	**0.002**
Gender						
Female	1 (reference)					
Male	1.356	[0.762, 2.414]	0.301			
BMI						
<25	1 (reference)			1 (reference)		
≥25	0.684	[0.467, 1.003]	**0.052**	0.649	[0.441, 0.957]	**0.029**
Gross hematuria						
No	1 (reference)					
Yes	1.413	[0.855, 2.333]	0.177			
T category						
Ta	1 (reference)			1 (reference)		
T1	1.662	[1.161, 2.379]	**0.006**	1.153	[0.771, 1.724]	0.489
Prior recurrence status						
Primary	1 (reference)			1 (reference)		
Recurrent	2.715	[1.816, 4.059]	**<0.001**	2.641	[1.753, 3.979]	**<0.001**
Pathology grade						
Low‐grade	1 (reference)			1 (reference)		
High‐grade	1.699	[1.154, 2.502]	**0.007**	1.528	[0.990, 2.359]	0.056
Tumor number						
Single	1 (reference)			1 (reference)		
Multiple	2.171	[1.463, 3.223]	**<0.001**	2.006	[1.340, 3.003]	**<0.001**

Abbreviations: BMI, body mass index = weight/height^2^; CI, confidence interval; HR, hazard ratio; SII, systemic immune‐inflammation index.

Bolded *p*‐values indicate statistically significant correlations (*p* < 0.05).

**TABLE 3:** Univariable and multivariable Cox regression analysis after propensity score matching

Variables	Univariable Cox analysis			Multivariable Cox analysis		
	HR	95% CI	*p*	HR	95% CI	*p*
Age (years)	0.991	[0.976, 1.006]	0.238			
Maximum tumor diameter (mm)	0.993	[0.976, 1.011]	0.46			
SII						
<505	1 (reference)			1 (reference)		
≥505	1.598	[1.048, 2.436]	**0.029**	1.646	[1.077, 2.515]	**0.021**
Gender						
Female	1 (reference)					
Male	1.278	[0.662, 2.467]	0.464			
BMI						
<25	1 (reference)					
≥25	0.734	[0.472, 1.142]	0.17			
Gross hematuria						
No	1 (reference)					
Yes	1.644	[0.895, 3.019]	0.109			
T category						
Ta	1 (reference)			1 (reference)		
T1	1.478	[0.978, 2.234]	**0.063**	1.107	[0.696, 1.762]	0.667
Prior recurrence status						
Primary	1 (reference)			1 (reference)		
Recurrent	2.376	[1.504, 3.754]	**<0.001**	2.475	[1.563, 3.918]	**<0.001**
Pathology grade						
Low‐grade	1 (reference)			1 (reference)		
High‐grade	1.858	[1.195, 2.891]	**0.006**	1.703	[1.038, 2.795]	**0.035**
Tumor number						
Single	1 (reference)			1 (reference)		
Multiple	1.871	[1.208, 2.899]	**0.005**	1.738	[1.116, 2.705]	**0.014**

Abbreviations: BMI, body mass index = weight/height^2^; CI, confidence interval; HR, hazard ratio; SII, systemic immune‐inflammation index.

## 4 DISCUSSION

In 2006, based on patients' T category, concurrent CIS, the number of tumors, prior recurrence status, maximum tumor diameter, and pathology grade, the European Organisation for Research and Treatment of Cancer (EORTC)^3^ published its first scoring system for predicting recurrence risks and progression in individual NMIBC patients. Moreover, CUETO^3^ proposed its scoring system after 3 years. Compared to EORTC, CUETO included risk factors such as gender and age, whereas it excluded tumor size. With the progress of related studies, more influential factors related to the prognosis of NMIBC patients have been proposed and applied to assess and predict patient prognosis.^7^

Mediators such as chemokines or cytokines induce an inflammatory state in the body and promote the proliferation and progression of tumor cells. Furthermore, activation of oncogenes can drive the carcinogenesis of an inflammatory state. Thus, inflammation and cancer are mutually reinforcing and closely associated.^8^, ^9^, ^12^, ^13^ SIR indicators are used to comprehensively evaluate subjects' inflammatory and nutritional status, which have been considered significant for the prognostic assessment of genitourinary malignancies.^22^ SII reflects the close relationship between serum platelets, neutrophils, and lymphocytes as a SIR indicator. It has been proposed as a potential factor in predicting survival outcomes in NMIBC patients.^23^, ^24^, ^25^, ^26^, ^27^ In multivariate analyses of previous studies, the HR of SII ranged from 1.23 to 2.127 when the survival endpoint of the study was RFS. However, when the survival endpoint was PFS, overall survival, and tumor‐specific survival, the HR of SII was 1.84, 2.104, and 1.716, respectively. However, none of the previous studies considered the potential selection bias and baseline imbalance bias. The present study demonstrated that SII (HR [95% CI]: 1.646 [1.077, 2.515], *p* = 0.021) is an independent predictor of RFS in NMIBC patients using 1:1 matched PSM analysis. Meanwhile, it could accurately stratify between high and low risk groups of poor RFS in the T1 stage (*p* = 0.025), primary status (*p* = 0.049), high grade (*p* = 0.0015), and multiple lesions (*p* = 0.043) subgroups. Elevated serum SII levels can be explained by a relative increase in platelet count and the neutrophil count or a relative decrease in lymphocyte count. Lymphocytes are crucial in host antitumor immunity as they mediate cytotoxic cell death and inhibit tumor cell proliferation and metastasis. A decrease in lymphocytes results in a decrease in the antitumor capacity of the internal environment, which increases the risk of cancer recurrence and progression.^28^, ^29^ Platelets promote the release of angiogenic factors,^30^, ^31^ which stimulate tumor angiogenesis and protect tumor cells from cytolysis. On the other hand, neutrophils secrete a large amount of vascular endothelial growth factors, which accelerate tumor angiogenesis and promote tumor carcinogenesis and metastasis.^32^, ^33^, ^34^

Most previous studies have concluded that elevated BMI is positively associated with the occurrence and poor prognosis of NMIBC.^35^, ^36^, ^37^, ^38^ In multivariate analyses of previous studies, the HR of SII ranged from 1.23 to 2.127 when the survival end point of the study was RFS. When the survival endpoint was PFS, the HR of SII was 1.84. When the survival endpoint was overall survival, the HR of SII was 2.104. When the survival end point of the study was tumor‐specific survival, the HR of SII was 1.716. However, in our unmatched Cox regression, elevated BMI was an independent protection factor in predicting RFS (HR [95% CI]: 0.649 [0.441, 0.957], *p* = 0.029). This finding supports a potential paradox in previous bladder cancer studies related to the effects of obesity. As per our understanding, this result may be related to the choice of the treatment regimen, which is consistent with the findings of Brooks et al.^39^ In 2019, Kim et al.^40^ first proposed gross hematuria as a potential predictor of NMIBC recurrence. However, in our study, gross hematuria was not statistically significant in the univariate regression, neither before nor after PSM.

The limitations of this study are inherent to its retrospective, observational design, and associated biases. First, our sample size warrants further expansion to validate the results. Second, identifying concurrent CIS is time‐consuming, and we are working with pathologists to incorporate this variable in a follow‐up study. Meanwhile, although the vast majority of patients included in the study received immediate single instillation within 24 h after TURBT, further control for consistency of patient regimens may have made more convincing conclusions. Lastly, the follow‐up period is too short, and the initial follow‐up time should be extended further.

## 5 CONCLUSION

In this retrospective study, the potential of SII for predicting RFS in patients with NMIBC was evaluated. To our knowledge, this is the first large sample study evaluating the prognostic value of SII in NMIBC with PSM. We proved that elevated SII was an independent risk factor for predicting RFS in NMIBC patients.

## AUTHOR CONTRIBUTIONS

**Li Ding:** Conceptualization (equal); writing – original draft (equal). **Xiangbu Wang:** Methodology (equal); validation (equal). **Xiaobin Deng:** Data curation (equal). **Wentao Xia:** Data curation (equal). **Kun Wang:** Data curation (equal). **Xianlin Yu:** Data curation (equal). **Yaotian Huang:** Data curation (equal). **Junqi Wang:** Conceptualization (lead); methodology (lead); validation (lead); writing – original draft (lead).

## FUNDING INFORMATION

This study was funded by the second round of Xuzhou Medical Leading Talents Training Project (XWRCHT20210027).

## CONFLICTS OF INTEREST

None of the authors have conflicts of interest to disclose. None of the authors have financial relationships relevant to this article to disclose.

## ETHICS APPROVAL AND CONSENT TO PARTICIPATE

This study was approved by the Ethics Committee of the Affiliated Hospital of Xuzhou Medical University (XYFT2022‐KL340‐01) and the Ethics Committee of the First Affiliated Hospital of Guangxi Medical University (2022‐E318‐01). The requirement for written informed consent has been accordingly waived due to the retrospective study design.

## CONSENT FOR PUBLICATION

Not applicable.

## Data Availability

Due to ethical restrictions, the raw data underlying this paper are available upon request to the corresponding author.
